# The oral cavity is not a primary source for implantable pacemaker or cardioverter defibrillator infections

**DOI:** 10.1186/1749-8090-8-73

**Published:** 2013-04-10

**Authors:** Jörg Eberhard, Nico Stumpp, Fadi Ismail, Ulrike Schnaidt, Wieland Heuer, Maximilian Pichlmaier, Christian Kühn, Axel Haverich, Meike Stiesch

**Affiliations:** 1 Department of Prosthetic Dentistry and Biomedical Material Sciences, Hannover Medical School, Carl-Neuberg-Str. 1, Hannover 30625, Germany; 2 Department of Cardiac-, Thoracic-, Transplantation- and Vascular Surgery, Hannover Medical School, Carl-Neuberg-Str. 1, Hannover 30625, Germany

**Keywords:** Biofilm, Infection, Pacemaker, Defibrillator, Oral cavity

## Abstract

**Background:**

To test the hypothesis that the oral cavity is a potential source for implantable pacemaker and cardioverter defibrillators infections, the bacterial diversity on explanted rhythm heart management devices was investigated and compared to the oral microbiome.

**Methods:**

A metagenomic approach was used to analyze the bacterial diversity on the surfaces of non-infected and infected pacemakers. The DNA from surfaces swaps of 24 non-infected and 23 infected pacemaker were isolated and subjected to bacterial-specific DNA amplification, single strand conformation polymorphism- (SSCP) and sequencing analysis. Species-specific primer sets were used to analyze for any correlation between bacterial diversity on pacemakers and in the oral cavity.

**Results:**

DNA of bacterial origin was detected in 21 cases on infected pacemakers and assigned to the bacterial phylotypes *Staphylococcus epidermidis*, *Propionibacterium acnes*, *Staphylococcus aureus*, *Staphylococcus schleiferi* and *Stapyhlococcus*. In 17 cases bacterial DNA was found on pacemakers with no clinical signs of infections. On the basis of the obtained sequence data, the phylotypes *Propionibacterium acnes*, *Staphylococcus* and an uncultured bacterium were identified. *Propionibacterium acnes* and *Staphylococcus epidermidis* were the only bacteria detected in pacemeaker (n = 25) and oral samples (n = 11).

**Conclusions:**

The frequency of the coincidental detection of bacteria on infected devices and in the oral cavity is low and the detected bacteria are highly abundant colonizers of non-oral human niches.

The transmission of oral bacteria to the lead or device of implantable pacemaker or cardioverter defibrillators is unlikely relevant for the pathogenesis of pacemaker or cardioverter defibrillators infections.

## Background

Today, the application of implantable pacemakers and cardioverter defibrillators (ICD) represents routine procedures in most hospitals. Because patients are often dependent on these artificial devices, their flawless function is vital. Nevertheless, complications do occur, the most frequent one being bacterial infection. Diagnosis and therapy of implant-associated infections are often difficult, protracted and expensive. Infections of implanted devices are associated with significantly increased morbidity and mortality and cause high health-care costs [1]. Early infections of pacemaker and ICD devices are usually caused by organisms which are introduced at the time of surgery and present most often with abscess formation or frank purulent wound dehiscence. On the other hand, late infections are the sequel of latent infections, in which the cause of the infection remains unresolved.

The oral cavity is a likely source of bacteria that may elicit infections on pacemaker and ICD devices after systemic transmission. Gingivitis and periodontitis are frequent chronic inflammatory processes belonging to the spectrum of periodontal diseases of the oral cavity affecting the tooth supporting tissues in response to bacterial accumulation. Constantly forming bacterial deposits on the teeth cause a chronic inflammatory response with many stages, ranging from reversible low-level inflammatory gingivitis to irreversible higher-level inflammatory periodontitis that is, if untreated, followed by tooth loss [2]. In addition to various epidemiological studies demonstrating a potential link between periodontitis and cardiovascular diseases it has been demonstrated that treatment of patients suffering from periodontitis reduces acute parameters of atherosclerosis and improves endothelial function [3]. During progression of gingival diseases, the epithelium becomes ulcerated to expose the underlying connective tissues and blood capillaries and facilitates entry of biofilm organisms or their products (e.g. bacterial heat-shock proteins) to the circulation [4]. Pathogens which have entered the bloodstream may adhere to the pacemaker lead that is located in the subcalvian vein on way to the right heart ventricle. Adherent bacteria may grow along the lead to the pacemaker, which is located in a connective tissue pouch for the initiation of an inflammatory reaction.

Therefore the aim of the study was to identify the bacterial composition of the oral cavity and implantable pacemaker and cardioverter defibrillator devices explanted with a diagnosis of infection, to test the hypothesis that the oral cavity is a potential source for pacemaker and ICD infections.

## Methods

### Sampling

From April 2005 to December 2010, rhythm management devices explanted from healthy patients and from patients suffering from pacemaker or ICD infection were collected. The time between implant or revision procedure and the diagnosis of infection was at least one month. The devices were transferred to a sterile container and were immediately stored at −80°C. Informed consent was obtained from all patients and the Institutional Review Committee at the Hannover Medical School approved the study (no. 5253).

### Device-related infections

Pacemaker and ICD infection was defined as suggested by Chamis et al. [5]. Clinical evidence of infection were local signs of inflammation around the generator pocket; including erythema, warmth, fluctuance, wound dehiscence, tenderness, purulent drainage or erosion. A diagnosis of infection was solely assigned to ICDs with a positive detection of bacteria by conventional culture techniques in routine laboratory diagnostics. Positive blood cultures as well as echocardiographically proven vegetations on the electrodes of the device were also considered as evidence for manifest infections. A pocket erosion was diagnosed when a lead and/or pulse generator perforated the skin. Impending or frank erosions without apparent infectious manifestations were not included among infectious complications. According to the local protocol single-shot antibiotics (Cefuroxim) were prescribed 30 minutes ahead of the surgical procedure in patients with non-infected pacemakers, patients with a diagnosis of an infected pacemaker used antibiotics at least 24 hours before the surgical intervention.

### Clinical oral examination and sampling of oral bacteria

All subjects underwent a clinical oral examination conducted by one examiner (F.I.) at the day before surgery. The amount of dental plaque attached to the tooth surfaces was recorded by the Plaque-index (PI) according to Sillness and Löe [6] at the mesio-buccal, buccal, disto-buccal, disto-oral, oral and mesio-oral sites of each teeth. Probing pocket depth (PPD) was recorded with a periodontal probe to identify the depth of the gingival sulcus, which is a measure of periodontal disease activity. The probing pocket depths measurements were also performed at 6 sites per tooth followed by recording of the bleeding frequency upon probing (BOP). This measurement is a highly sensitive marker for the inflammatory reaction within the gingival or periodontal sulcus.

Plaque samples were collected using sterile filter papers from four sites of the tooth showing the deepest probing pocket depth measurements. The samples were taken at tooth sites and not at the or other mucous tissues, because inflamed gingival tissues are most relevant for the spread of bacteria or inflammatory mediators into the bloodstream. Prior to sampling, the tooth surfaces were isolated by cotton rolls and gently dried with an air syringe. Paper points were carefully placed into the gingival sulcus for 30 sec to collect plaque and were afterwards placed into a 1.5 ml Eppendorf tube and kept at −80°C until analysis. Paper points that were contaminated with blood were discarded.

### DNA Isolation, amplification and SSCP analysis

For the analysis of device related bacteria the attached microorganisms were scraped of the ICD surfaces in a clean air cabinet by use of sterile scalpel blades. Total genomic DNA (deoxyribonucleic acid) was isolated using the QIAamp DNA Mini Kit (Qiagen) according to the manufacturer’s protocol for positive bacteria. This protocol was used to effectively breakup the bacterial cell wall of Gram+ and bacteria. A mechanical disruption step with a bead mill (Precellys 24; Bertin Technologies) was performed prior to spin-column DNA extraction. An approximately 500 bp (base pair) fragment of the 16S rDNA (ribosomal deoxyribonucleic acid) was amplified using the universal primers 27f and the 5^′^-prime phosphorylated 521revP. PCR (polymerase chain reaction) was conducted on a TProfessional thermocycler (Biometra) following a 32 cycles standard PCR protocol. Amplicons were purified over silica spin-columns (QIAquick PCR Purification kit; Qiagen) and dried overnight at 40°C.

Single strand conformation polymorphism (SSCP) analyses were performed on a DCode Universal Mutation Detection System (Bio-Rad). The DNA fragments were electrophoretically separated on a 10% polyacrylamide gel at 350V (20°C) for 24 h in 1x TBE buffer (Bio-Rad). DNA bands were visualized by silver-staining (Silver-Stain kit; Bio-Rad). Bands were excised and DNA was extracted and reamplified. PCR products were purified and subsequently sequenced (Seqlab). Obtained sequences were processed using the BioEdit software package (v7.0.9, Ibis Biosciences) and compared to GenBank database sequences from the National Center for Biotechnology Information (NCBI). For identification of the closest match the NCBI Basic Local Alignment Search Tool [7] as wells as the SEQMATCH and CLASSIFIER tools from the Ribosomal Database Project [8] were used.

Genomic DNA from oral plaque was isolated according to our protocol and PCR-analyzed for any correlation with bacteria found on ICD. Therefore species-species primer sets for *Propionibacterium acnes*, *Staphylococcus epidermidis* and *Staphyolococcus aureus* were synthesized (Eurogentec) and the PCR amplification was conducted with 5 μl of isolated genomic DNA and 40 amplification cycles as described elsewhere [9,10]. Five μl of each amplification reaction were analyzed on an agarose gel (Agarose MP, Applichem) and rated as positive detection when a clear single band of the expected size was recognizable after ethidium bromide staining.

### Statistical analysis

Mean and standard deviations were calculated for all clinical and laboratory parameters with the patient as the statistical unit. For the comparison of intergroup differences the Wilcoxon signed-rank test was used. Differences were reported significant for *P*-values ≤ 0.05.

## Results

### Patient characteristics

Twenty-three patients with a diagnosis of pacemaker or ICD infection (age: 63.5 ± 19.3 years, 15 males) and 24 patients with no signs of clinical infection (age: 73.3 ± 11.6 years, 15 males) were enrolled (Table 1). In the non-infected pacemaker group 2 patients had a diagnosis of diabetes, 8 were current smokers, in the infected pacemaker group 1 patient suffered from diabetes and 6 were current smokers. In both groups no patient took any immunosuppressive medication. During and following device and lead extraction no deaths occurred and no serious complications were encountered.

**Table 1:** Baseline characteristics of the patients

**Characteristic**	**Non-infected**	**Infected ICD**	***P*-value**
Age - yr	63.5 ± 19.3	73.3 ± 11.6	0.109
Male sex - no. (%)	15 (65)	15 (65)	
Hypertonic - no. (%)	9 (39)	11 (49)	
Diabetes - no. (%)	2 (8.7)	1 (5)	
Current smoking - no. (%)	8 (34.8)	6 (26)	
Immunesupressive medication - no. (%)	0 (0)	0 (0)	
**Lead infection – no.**		**18**	
**and/or pocket infection – no.**		**14**	
**and/or bacteremia – no.**		**5**	
**and/or endocarditis – no.**		**1**	
BOP - % (min-max)	30 (0–62)	23 (0–68)	0.914
PI - mean (min-max)	1.19 (0.16-2.66)	1.35 (0.75-3.0)	0.004
PPD - mm (min-max)	4.20 (1.55-9.09)	3.11 (2.78-6.37)	0.588
Number of teeth - no. (min-max)	17.4 (0–31)	11.2 (0–27)	0.114
ICD for - years	7.5 (4–9)	2.7 (0–9)	0.006

All patients with non-infected pacemaker were scheduled for aggregate changes at least > 12 months after implantation. Of the 23 patients with infected pacemaker, 0 (0%) had an early infection (<1 month after procedure), 4 (17.4%) a late infection (1–12 months after the device-related procedure) and 19 (82.6%) a delayed infection (>12 months after the procedure).

The mean time delay from device implantation or revision to surgery was 90 months in the non-infected group, in the infected group this time period was 32 months (*P* = 0.006).

Routine bacterial culturing verified basically *Staphylococcus* and *Propionibacterium acnes* on leads (n = 18) and/or in device pockets (n = 14). A bacteremia was diagnosed in 5 cases and one patient suffered from bacterial endocarditis (*Staphylococcus aureus*).

### Oral health status

None of the patients had any tooth extraction, periodontal therapy or surgical intervention 6 months prior to the pacemaker removal. The plaque index values of 1.19 PI respectively 1.35 PI were measured for the non-infected and infected pacemaker groups, which is characteristic for a good to average level of oral hygiene. The differences were statistically significant (*P* = 0.004). Mean probing pocket depths of 4.2 mm were recorded in patients with non-infected pacemaker in contrast to patients with infected pacemaker that showed a mean probing pocket depth value of 3.1 mm. The differences were statistically not significant (*P* = 0.588). Bleeding on probing showed a value of 30% in patients with non-infected pacemaker and a value of 23% in patients with infected pacemaker (*P* = 0.914). In accordance to the plaque index, the patients showed average bleeding frequencies. Patients in the non-infected pacemaker group had a mean number of teeth of 17.4, the patients in the infected pacemaker group had 11.2 teeth (*P* = 0.114).

#### Microbiology

**Figure 1:**
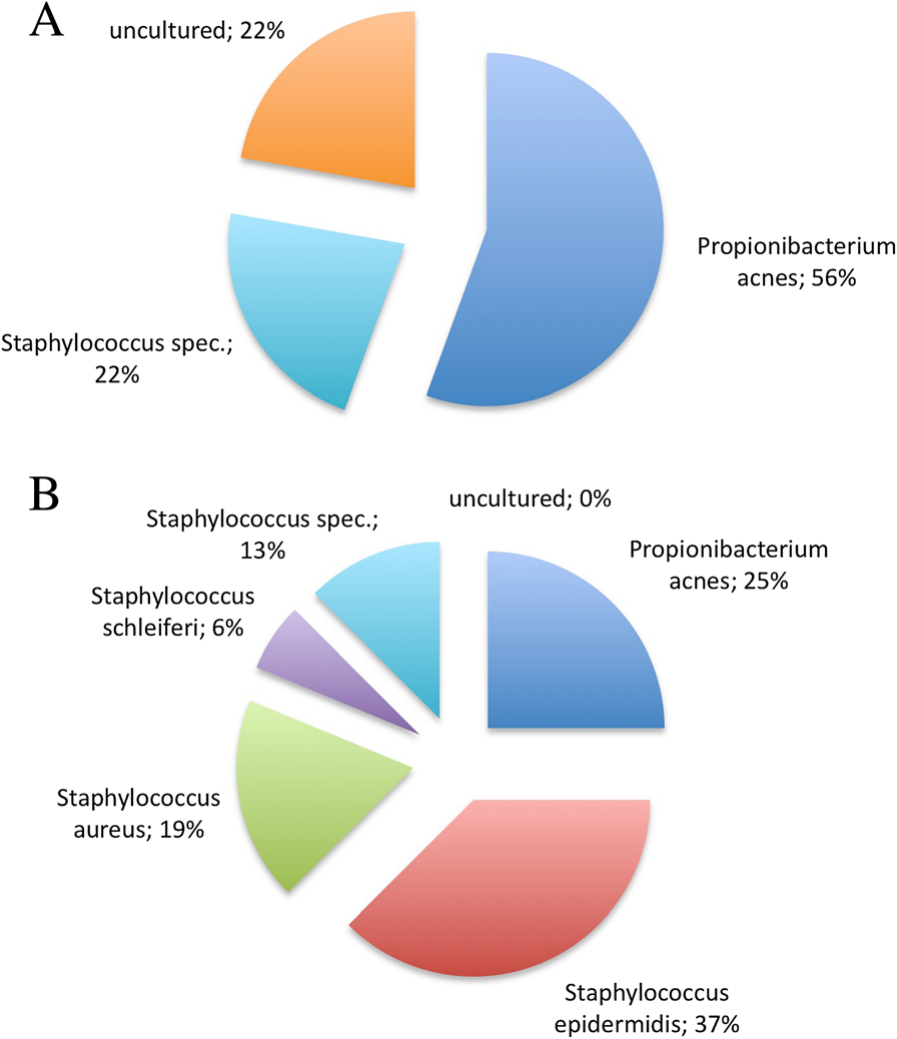
**Microbial findings at analyzed implants.** The pie charts depict the frequency of bacteria identified on (**A**) non-infected and (**B**) infected pacemaker devices.

In patients with non-infected ICDs (n = 24) bacterial DNA was isolated in 17 (70.9%) cases, out of these in 7 patients (29.2%) microorganisms were yielded by sequencing (Figure 1A). In 1 (4.2%) patient sequencing yielded polymicrobial results. Sequences from 5 (21.7%) patients yielded *Propionibacterium acnes*, 2 (8.7%) patients had *Staphylococcus sp*. and 2 (8.7%) patients had uncultured bacteria. In all patients with non-infected ICD that were positive for *Propionibacterium acnes* or *Staphylococcus* the species-specific primers also identified the respective microorganisms in oral plaques (Table 2).

**Table 2:** Detection frequencies of bacteria

**Bacterial species**	**Detection frequency (n)**			
	**RMD clinically non-infected (n = 24)**		**RMD clinically infected (n = 23)**	
	**device**	**oral**	**device**	**oral**
*Propionibacterium acnes*	5	5	4	1
*Staphylococcus epidermidis*			6	3
*Staphylococcus aureus*			3	
*Staphylococcus schleiferi*			1	
*Staphylococcus* spec.	2	2	2	
*uncultured*	2			

In patients with infected pacemakers (n = 23) bacterial DNA was isolated in 21 (91.3%) cases and in 15 patients (65.2%) sequencing yielded microorganisms (Figure 1B). In 4 (17.4%) patients sequencing yielded polymicrobial results. Sequences from 6 (26.1%) patients yielded *Staphylococcus epidermidis*, 4 (17.4%) patients had *Propionobacterium acnes*, 3 (13.0%) patients *Staphylococcus aureus*, 2 (8.7%) patients had *Staphylococcus* and 1 (4.3%) patient had *Staphylococcus schleiferi*. One out of 4 patients with a positive detection of *Propionibacterium acnes* on infected pacemaker, was also positive for this microorganism in dental plaque. *Staphylococcus epidermidis* was identified in the oral plaque samples of 3 patients.

## Discussion

Implantations of cardiac pacemakers and cardioverter defibrillators have become indispensable techniques in medical treatment for decreasing the mortality in patients with arrhythmias or cardiomyopathy [11]. However, infection rates range from 1-12% and can involve the pacemaker/ICD pocket, electrodes, generator or endocardium [12]. Aside from difficulties to diagnose infections of cardiac devices the classification according to the time course after implantation, similar to prosthetic valve endocarditis (PVE), becomes a useful tool to arrange temporary transvenous pacemaker (TVPM)/ICD infections [13,14]. Early infection again occurs as a result of intraoperative contamination and tends to be predominated by *Staphylococcus aureus*. Late infection is usually a result of cutaneous erosion of the generator. Microorganisms from the pocket can spread along the electrodes to endocardial surfaces, including the tricuspid valve. An alternative way for late infections includes hematogenous seeding of the endovascular electrode during transient bacteremia from a distant site of infection. Therefore the most common pathogen is *Staphylococcus aureus* and other less commonly identified pathogens, including viridans group streptococci, enterococci and gram-negative bacilli [5].

The diagnosis of pacemaker and ICD infections should be suspected in patients carrying such devices with unexplained fever. These intravascular device infections can be diagnosed through serologic, radiologic, and echocardiographic measurements. The clinical manifestations are variable and depend on the affected component and vary from slight pocket edema with or without erythema, pain and warmth to fever and abscess formation [15]. The optimal management of pacemaker and ICD infections seems to be a combined medical/surgical approach consisting of complex device removal/re-insertion with prolonged antibiotic therapy.

In cases of device infection in combination with skin perforation the diagnosis is easy, however, the exact timepoint of infection remains unclear. It is possible that a former infection lead to the accumulation of bacteria followed by the skin passage or the skin passage is followed by bacterial migration into the device pouch. Pacemakers that were removed during routine inspections were contaminated with bacterial DNA with a frequency of approximately 70%. Especially in these cases the present study was intended to identify the oral cavity as a likely source of bacterial contamination of pacemaker.

Several epidemiological data have been published that clearly identify chronic inflammatory diseases of the oral cavity, in particular periodontitis, as a risk factor for various systemic conditions, e.g. cardiovascular diseases, stroke, preterm low birth weight and diabetes [16]. Periodontal diseases are a candidate group of extravascular diseases with adverse systemic effects, because of their chronic and painless character, combined with extensive amounts of bacteria and ulcerative surfaces, which are often a source for a systemic dissemination of inflammatory products and bacteria into blood [17]. This transmission may be spontaneously, during eating or toothbrushing. The transfer of oral bacteria has been proofen e.g. by the detection of oral pathogens as well as commensal bacteria within atheromatous plaques [18,19]. Especially bacteria associated with periodontitis like *Porphyromonas gingivalis* are in the focus of interest, because of several virulence mechanisms that are relevant for the pathogenesis of atherosclerosis [20].

Here we used 16S rDNA based metagenomics to evaluate the bacterial composition of non-infected and infected pacemakers. This approach allowed a relatively comprehensive description of dominant microbial communities associated with pacemakers. The detection of bacteria on asymptomatic rhythm heart management devices removed for battery exchange has been shown [21], the present study was aimed to identify the oral cavity as a potential source for bacteria colonizing non-infected and infected devices. We found that non-infected pacemakers are frequently contaminated with bacteria and if bacteria were found, skin microorganisms were dominating. Infected pacemakers were prevalently colonized by *Propionibcterium acnes* and *Staphylococcus* where *Staphylococcus epidermidis* was most often detected. If bacteria were identified on pacemaker they were only irregularly isolated in the oral cavity. The oral, respectively periodontal health condition of patients with non-infected and infected pacemakers showed no remarkable difference. From the present study the oral cavity could be excluded with high certainty as a bacterial source for late pacemaker and ICD infections. Although, the present study simply address bacteria located at the gingival margin, bacteria colonizing other oral niches are unlikely relevant for inflammatory process induced by bacteremia. These bacteria become swallowed on a regular basis and do not have access to the bloodstream.

The present study describes for the first time the microbiota of a patient group with non-infected or infected pacemakers using the SSCP technique. In contrast to methods using specific 16S rDNA primers the SSCP method is aimed to detect diverse dominant bacterial genera or species in a biofilm consortium with a detection limit of approximately 1000 CFU/ml (colony forming units/ml) as determined in a series of preliminary experiments (data not presented). To identify bacterial consortia in diverse habitats a metagenomic approach is helpful, because of the potential identification of bacteria, irrespective of their taxonomical classification, which is not possible by searching for known genera or species. For the present study no state-of-the-art whole-microbiome sequencing method was used. In contrast to pyrosequencing techniques that may detect even single copies of individual 16s rRNA gene copies, SSCP analysis detects dominant bacterial species in biofilm community. This approach is more suitable to identify bacteria that initiate inflammatory processes. Although different antibiotic regimes were used in the test and control groups, the bacterial biofilm composition could be comprehensively analyzed by SSCP analyses, because detection does not rely on viability.

Chua et al. [14] found that 42% of all patients with pacemakers had a delayed infection and it is of fundamental interest to reveal how these infections occur. Local perioperative wound contamination and colonisation by haematogenous routes are the two most advocated mechanisms, but none has been fully validated. *Propionibacterium acnes* and *Staphylococcus* were present in a frequent number of non-infected pacemaker specimens and represent bacteria commonly found on skin. It is likely that pacemakers are contaminated by these microorganisms during the surgical procedure. In a previous publication Pichlmaier et al. (2008) found *Pseudomonas* (16%), *Staphylococcus* (11%), *Stenotrophomonas* (10%), *Rhizobium* (9%) and *Propionibacterium* (7%) most commonly in asymptomatic rhythm management devices. This spectrum of bacteria is very different from that commonly found in infected pacemakers, however, overgrowth of opportunistic bacteria and a concomitant decrease of commensal bacteria is a plausible explanation [22]. Da Costa et al. [23] strongly supported the hypothesis that pacemaker-related infections are mainly the result of local contamination during implantation. They found that patients with delayed infections (16–29 months) had the same strain of microorganisms cultured from infected device pockets as was isolated from the skin and pocket at device implantation. Also, the fact that nearly all cultured microorganisms are part of the skin flora supports the hypothesis of wound contamination during the device procedure. The data of the present study support this mode of infection.

For infected pacemakers a more diverse microbiota was identified, however, again commensal skin bacteria were most frequently detected. *Staphylococcus epidermidis* was most frequently detected on pacemakers with a diagnosis of inflammation and is known to be responsible for severe nosocomial infections. Recently, the microbiology of cardiac device infections were investigated by culture and in this study *Staphylococcus aureus* and coagulase-negative staphylococci were most frequently isolated [24]. In the present study no gram-negative bacteria were identified, which is in contrast to publications that report gram-negative bacteria in 4-15% of the specimens [14,25]. A lower sensitivity to gram-negative bacteria of the used universal primers may be an explanation. *Propionibacterium acnes* and *Staphylococcus aureus* were identified in 4 out of 10 cases in dental plaque using specific primers after they have been identified on infected pacemakers. These bacteria belong to the normal oral microbiota according to the HOMD database (http://www.homd.org). It may be possible that these bacteria have been transferred from the oral cavity to the generator leads and pacemaker to initiate infection, however, the low frequency observed and the low number of these bacteria generally found in the oral cavity makes this imagine unlikely. The observation that non-infected pacemakers and infected pacemakers are most frequently contaminated with skin pathogens makes it likely that they were incooperated during surgery and initiate infection after a latent period within the tissue pouch.

One intriguing observation was the similar periodontal health condition between patients with non-infected and infected pacemakers. Sensitive parameters characterizing the previous and actual periodontal disease activity were recorded and no significant differences were observed. While the parameter BOP accurately determines the actual inflammatory reaction of the periodontal tissues, the number of residual teeth enables a good impression of disease activity through live, because teeth were lost in elderly most often due to periodontitis. In 2005 in Germany elderly between 65 and 74 years had approximately 14.2 teeth, which is in good agreement to the data of the two groups enrolled in the present study [26]. From the present data it could not be excluded that the low number of teeth in the infected pacemaker group is a result of extensive tooth extractions due to the repeated surgical interventions. In accordance to several publications analyzing risk factors for pacemaker and ICD infections the present study identified no correlation between pacemaker infection and oral or periodontal health conditions of the patient [24,27]. However, periodontitis is a proven risk factor for cardiovascular diseases and any patient with an incooperated pacemakers or ICDs should avoid any inflammatory reaction of the oral tissues affecting the systemic inflammatory burden.

## Conclusions

In summary, the transmission of oral bacteria to the lead or device of implantable pacemakers or cardioverter defibrillators is unlikely relevant for the pathogenesis of pacemaker and ICD infections, because of the low frequency of the coincidental detection of bacteria on infected devices and in the oral cavity and because the detected bacteria are highly abundant colonizers of non-oral human niches. Even clinical non-infected pacemaker devices were contaminated with relevant amounts of bacterial biofilms.

## Abbreviations

BOP: Bleeding frequency upon probing; bp: Base pair; CFU: Colony forming units; DNA: Deoxyribonucleic acid; ICD: Implantable pacemakers and cardioverter defibrillators; NCBI: National center for biotechnology information; PCR: Polymerase chain reaction; PI: Plaque index; PPD: Probing pocket depth; PVE: Prosthetic valve endocarditis; rDNA: Ribosomal deoxyribonucleic acid; SSCP: Single strand conformation polymorphism; TVPM: Temporary transvenous pacemaker.

## Competing interests

The authors declare that they have no competing interests.

## Authors’ contributions

JE, CK, AH and MS participated in the design of the study, interpreted the data and drafted the manuscript. All authors read and approved the manuscript. NS did the microbiological analysis and US, FI, WH and MP have made substantial contributions to data acquisition and clinical sampling. All authors read and approved the final manuscript.
